# Analysis of Vocal Signatures of COVID-19 in Cough Sounds: A Newer Diagnostic Approach Using Artificial Intelligence

**DOI:** 10.7759/cureus.56412

**Published:** 2024-03-18

**Authors:** Bhavesh Modi, Manika Sharma, Harsh Hemani, Hemant Joshi, Prashant Kumar, Sakthivel Narayanan, Rima Shah

**Affiliations:** 1 Department of Community and Family Medicine, All India Institute of Medical Sciences, Rajkot, IND; 2 Department of Atomic Energy, Institute of Plasma Research, Gandhinagar, IND; 3 Department of Atomic Energy, Bhabha Atomic Research Centre, Visakhapatnam, IND; 4 Department of Pharmacology, All India Institute of Medical Sciences, Rajkot, IND

**Keywords:** ft-st, diagnosis of covid-19, screening of covid-19, artificial intelligence, covid-19

## Abstract

Background: Artificial intelligence (AI) based models are explored increasingly in the medical field. The highly contagious pandemic of coronavirus disease 2019 (COVID-19) affected the world and availability of diagnostic tools high resolution computed tomography (HRCT) and/or real-time reverse transcriptase-polymerase chain reaction (RTPCR) was very limited, costly and time consuming. Therefore, the use of AI in COVID-19 for diagnosis using cough sounds can be efficacious and cost effective for screening in clinic or hospital and help in early diagnosis and further management of patients.

Objectives: To develop an accurate and fast voice-processing AI software to determine voice-based signatures in discriminating COVID-19 and non-COVID-19 cough sounds for screening of COVID-19.

Methodology: A prospective study involving 117 patients was performed based on online and/or offline voice data collection of cough sounds of COVID-19 patients in isolation ward of a tertiary care teaching hospital and non-COVID-19 participants using a smart phone. A website-based AI software was developed to identify the cough sounds as COVID-19 or non-COVID-19. The data were divided into three segments including training set, validation set and test set. A pre-processing algorithm was utilized and combined with Short Time Fourier Transform feature representation and Logistic regression model. A precise software was used to identify vocal signatures and K-fold cross validation was carried out.

Result: A total of 117 audio recordings of cough sounds were collected through the developed website after inclusion-exclusion criteria out of which 52 have been marked belonging to COVID-19 positive, while 65 were marked as COVID-19 negative/unsure /never had COVID-19, which were assumed to be COVID-19 negative based on RT-PCR test results. The mean and standard error values for the accuracies attained at the end of each experiment in training, validation and testing set were found to be 67.34%±0.22, 58.57%±1.11 and 64.60%±1.79 respectively. The weight values were found to be positive which were contributing towards predicting the samples as COVID-19 positive with large spikes around 7.5 kHz, 7.8 kHz, 8.6 kHz and 11 kHz which can be used for classification.

Conclusion: The proposed AI based approach can be a helpful screening tool for COVID-19 using vocal sounds of cough. It can help the health system by reducing the cost burden and improving overall diagnosis and management of the disease.

## Introduction

The new mutant strain of coronavirus, leading to coronavirus disease 2019 (COVID-19), originated in Wuhan and has spread rapidly across the world and the World Health Organization (WHO) declared it to be a pandemic. Globally, by mid-December 2022, there were 646,740,524 confirmed cases of COVID-19, including 6,637,512 deaths, reported to WHO [1,2]. Due to high population density, the rapidness of spread is predicted to be very high in India. In India till 15th December 2022, 44475647 cases and 530663 deaths had occurred due to COVID-19 as per reporting by WHO [2]. This highly contagious pandemic has significantly affected the overall quality of life of the patients. In early days of pandemic, only two diagnostic techniques were used for detection of COVID-19. One was viral nucleic acid detection using real-time polymerase chain reaction (RT-PCR) and other was high resolution CT scan (HRCT) but among them RT-PCR was widely used. However, both of the above diagnostic tools were costly, limited availability and required expertise and in a day limited numbers of cases can be diagnosed through RT-PCR and HRCT [3,4]. Moreover, both the tests are costly giving a huge cost burden on health care system [5].

Tremendous advancements in digitalization have introduced integration of Artificial Intelligence (AI) in medical diagnosis field also. Artificial intelligence (AI) works on algorithms and a proper database integrated algorithm can not only predict potential epidemics but even show a proper pathway in diagnosing certain challenging diseases. AI nowadays finds its immense applicability in the field of medicine especially in respiratory medicine. Machine Learning (ML) can be used by collecting different cough sounds and design an automated application-based process which can be used in the field of respiratory disease to diagnose suspected COVID-19 patients. The proposed voice processing AI solution promises a fast and easy-to-deploy screening mechanism in the field with very low cost and larger availability [6,7].

There is a need for development of specific and accurate screening tool that can detect COVID-19 easily provided that the tool is quick, easy to administer and cost effective. Most cases of morbidity and mortality of COVID-19 are usually presented in the late stage of the disease at the hospital, and delay in diagnosis is identified as one of the factors affecting the overall outcome of the disease [8]. Though this tool would not help in replacing the differential medical diagnosis of COVID-19 through laboratory testing, but still would play a very vital role in identifying suspects, differential diagnosis and reducing the burden on existing system.

With this study, we aim to find voice-based signatures for discriminating between COVID-19 and non-COVID-19 cough sounds. These signatures can be utilized for remote screening for COVID-19, which will be a great help to health care system and which will be a less expensive solution for screening large number of people. Considering all these factors, primary aim of this study was to develop a mobile based application by identification and determination of vocal signatures that will help in screening for COVID-19 in the field which is fast and accurate.

## Materials and methods

This is a prospective study with an innovative design involving online/offline voice data collection of COVID-19 and non-COVID-19 patients using smartphones and generating an AI-based mechanism for diagnosis of COVID-19.

Sample size

The study was conducted from March to May 2020. Considering the prevalence of COVID-19 first wave in India during the study period, a total of 117 data, of which 52 were from patients and 65 from non-patients were collected. The data was further divided into a training set (42 patients and 55 non-patients), a validation set (five patients and five non-patients), and a test set (five patients and five non-patients). Voice data was collected from COVID-19 patients (either based on HRCT findings or RT PCR testing) who were admitted/discharged from across the state, while voice data from non-patients was collected from students, staff, and volunteers using a smartphone.

Participant selection

Adult patients with COVID-19 diagnosed by the physician (either based on HRCT findings or RT-PCR testing) and gave consent were included in the study and were requested to record six different types of voice samples on a smartphone. Sounds recorded were the sample of cough sounds, Vowel aaa, Vowel eee, Vowel ooo, counting from 1 to 20 in English, and the English alphabet A to Z. For control or non-patient group people who do not have COVID-19 with the presence or absence of symptoms such as cough, fever, etc. and who were able to provide above mentioned voice recordings were enrolled. Data was recorded preferably on a person's own smartphone. This provided sufficient sensor diversity to prevent model biasing. It was recommended that data from patients and non-patients follow similar distributions in age and gender. People who had difficulty speaking or who suffered from throat or respiratory conditions affecting normal speech other than COVID-19 were excluded from participation.

Randomization and blinding

The software utilized only the voice data and patient/non-patient status for searching vocal signatures related to COVID-19. K-fold cross-validation [9] was carried out by randomly selecting samples for training, validation, and testing during our study.

Ethical issues

Institutional Ethical Committee approval for the protocol was received from the Institutional Ethics Committee (IEC), Gujarat Medical Education & Research Society (GMERS) Medical College Gandhinagar, Gujarat. Written informed consent was obtained from all participants before the collection of data. This study involved the collection of personal information such as age, gender, COVID-19 status, and voice samples. The personal data was collected over an encrypted medium and was completely anonymized before processing. De-identified data were used for the development of the application. Strict confidentiality was maintained and the data were not shared outside the organizations concerned.

Process of data collection

**Figure 1:**
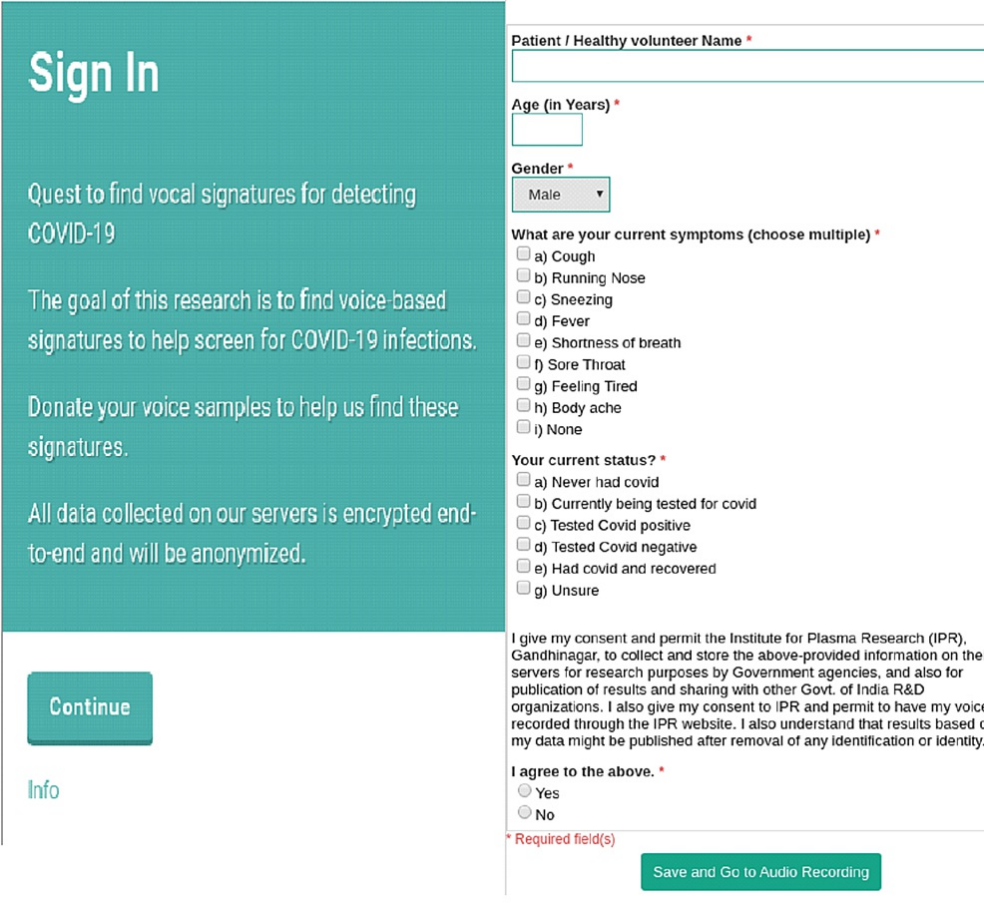
The web-based mechanism to upload data

This study involved the collection of personal information such as age, gender, COVID-19 status, and voice samples. A mechanism was developed for remote data collection and receiving them on our server using AI. Each user was required to record audio of three coughs. Patients were instructed to record audios in silent environments, however some had considerable amount of background noise. To extract only a clean coughing sound for further analysis, all audio files were pre-processed using Audacity® (a standalone, free & open-source audio processing software; www.audacityteam.org). Audio files were recorded by website users using either their smartphone mic or an external mic connected to a laptop or a personal computer (PC). After data recording, in order to help doctors/medical professionals/volunteers/patients, an easy-to-use web-based mechanism was developed. One of the key benefits of this mechanism was that it could be accessed using a mobile, laptop, or PC, and the upload of data could be done in the following three easy steps on the website (Figure 1). The steps are: 1) Go to the website and click on continue and start buttons to reach the questionnaire. 2) Answer a few questions related to your symptoms/health and give your consent to save your data on the Institute of Plasma Research (IPR) servers and to be used for research and development purposes. It assures that the data will be anonymized and no personal data will be stored. 3) Record the coughing sounds and voice samples, examples are also given to help recording. 4) Once done, the user can read the declaration carefully and then click on submit to upload the data.

Pre-processing of data

The steps used for pre-processing of data include a) manual selection of noise part of audio and performing noise reduction using Audacity. For very noisy audio, this step was repeated multiple times until no background noise could be heard. b) If the number of coughing sounds was less than three (which was the case for seven recordings), additional cough sounds were added by repeating the audio from the beginning. d) All silent parts of the audio were trimmed to 0.1 seconds. f) Any other background sound (e.g.: speech, breathing sound) was removed. g) Audio amplitude was normalized, and the signal was resampled at 48000 Hz before saving to disk.

Statistical analysis

A sophisticated pre-processing algorithm was utilized and combined with Short Time Fourier Transform feature representation (STFT) [10], and a logistic regression model [11] was used for analysis. The AI model was trained using an adaptive moment estimation (ADAM) gradient descent algorithm [12] and Principal Component Analysis [13] (PCA) was used for exploration.

Model development

**Figure 2:**
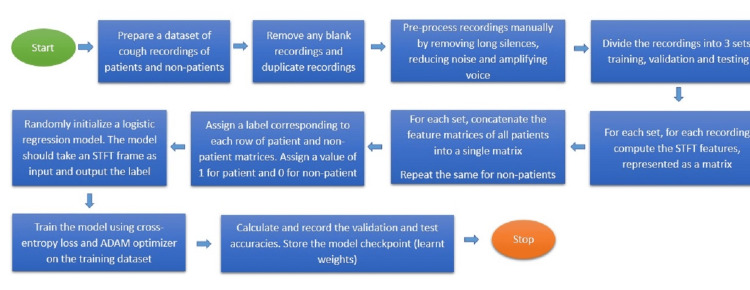
Flowchart of model training algorithm

The protocol for training, validation, and test sets: A standard machine learning framework was developed and used, wherein, independent samples (belonging to different speakers, recorded separately and at different times) were used to create three sets of data: training set (for learning the model), validation set (for comparing multiple models trained by same training set) and test set (for estimating real-world performance). A total of 117 (52 +ve and 65 -ve) samples of cough sounds were present in the database, wherein a training set comprising 97 (42 +ve and 55 -ve) samples, validation set, and test sets with 10 (five +ve and five-ve) samples were prepared. To get a reasonable estimate of model performance, experiments were repeated several times using different partitions for which elements in each set (separately for +ve and -ve categories) were selected uniformly at random for each experiment. Figure 2 provides the detailed method of the model training algorithm.

## Results

AI-based model development

Description of the data set obtained: A total of 141 audio recordings of cough sounds were collected through the developed website. Associated metadata such as name, age, gender, a list of COVID-19-related symptoms, and present COVID-19-related status were also collected. Out of 141 recordings, 66 have been marked which belong to COVID-19 positive, while 75 have been marked as COVID-19 negative/unsure/never had COVID-19, which were assumed to be COVID-19-negative for simplicity. After manually removing unnecessary data and duplicates (judging from corresponding name, gender, and age), 52 recordings belonging to the COVID-19 positive category and 65 recordings belonging to the COVID-19 negative category were compiled. The resultant dataset containing 117 recordings was then used for the analysis and model development.

Exploratory data analysis (EDA)

**Figure 3:**
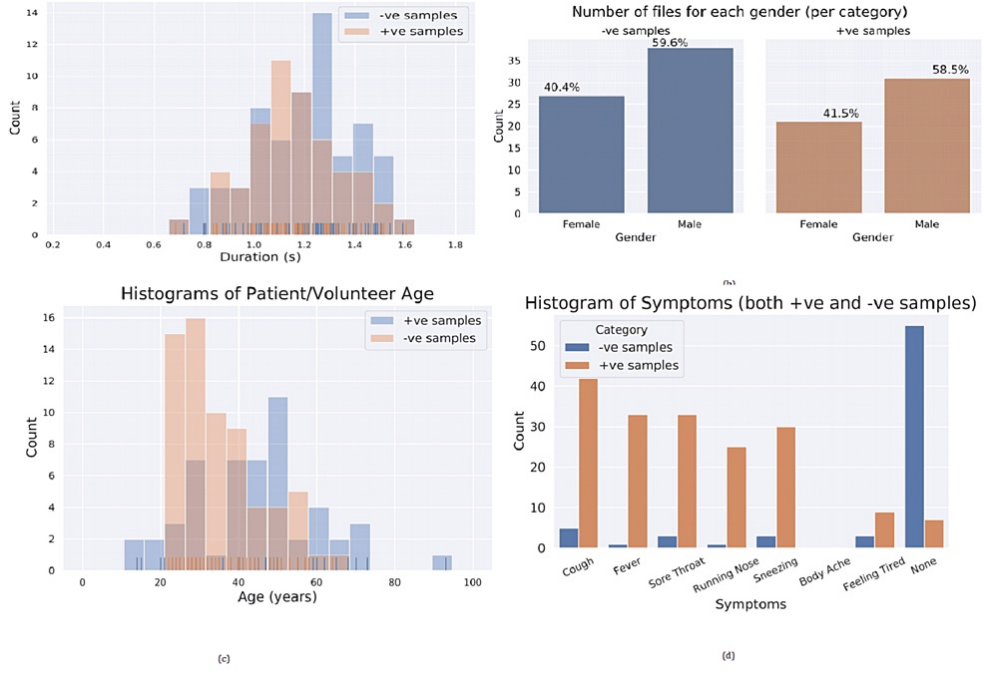
Histograms of study data (a) Histogram of cough file durations for COVID-19 positive and rest files (b) Number of files for each gender (separately shown for positive and negative samples). Please note that while the number of files between categories is different, the distribution is similar. (c) Histogram of patient/volunteer age. Please note that, unlike duration and gender, distribution is quite different between COVID-19 positive and negative samples. (d) Histogram of symptoms (separately for positive and negative samples). Please note that most of the negative samples were recorded by people with no symptoms.

Out of 141 total recordings collected, after removing the duplicates and unnecessary files, a total of 117 files (52 COVID-19 positives + 65 non-COVID-19) were included for analysis. Statistical analysis of audio file durations, speaker gender, speaker age and symptoms were done and are presented through appropriate charts (Figure 3). Duration of cough sounds recorded ranged from 0.7 seconds to 1.7 seconds, with the highest number of patients 1.2-1.4 sec duration. In COVID-19 and non-COVID-19 groups. No significant difference was found in the gender distribution of all enrolled patients. It was well evident from the statistical analysis and figures that the distributions of audio duration and gender were very similar between both categories. On the other hand, the distributions for age and symptoms were quite different. Such differences can lead to model biases where the model helps to distinguish between the categories based on a different factor (for example, age or symptoms). Such biases were eliminated by acquiring more data and preparing a balanced dataset. As a large amount of data was acquired, it was decided to perform a more systematic study with a balanced dataset.

Feature extraction and target label

**Figure 4:**
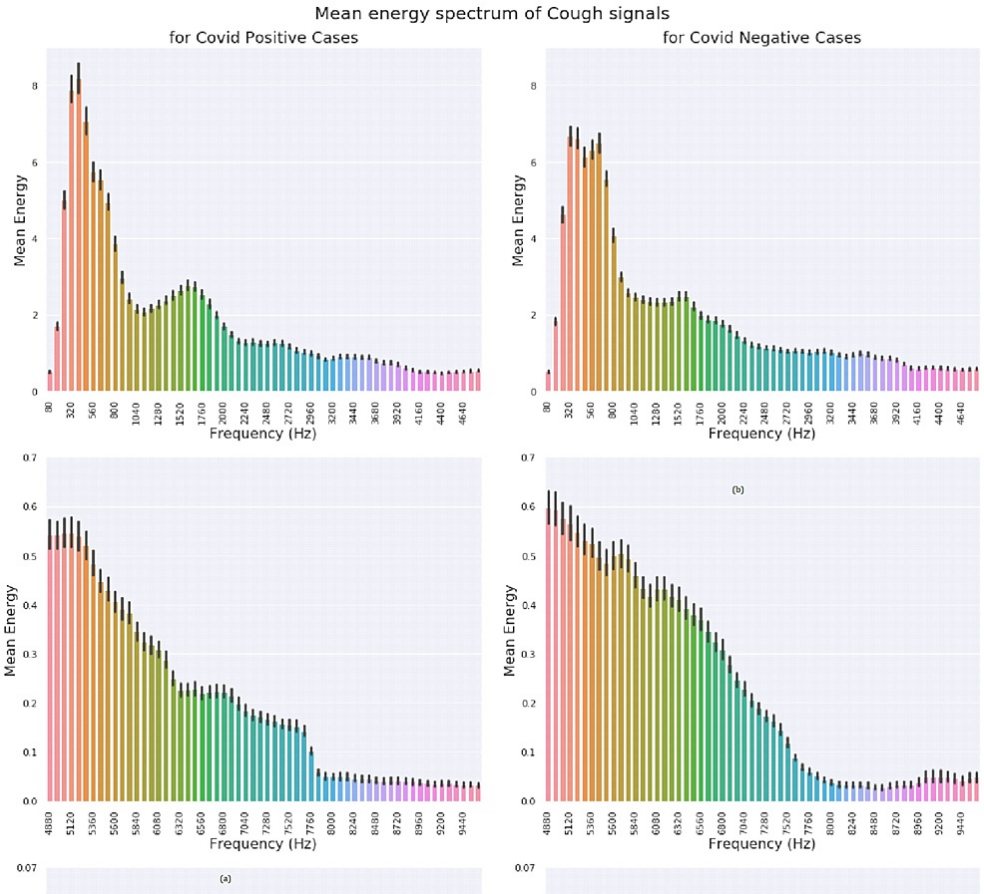
Mean energy spectrum of the recorded cough sound signals (a) COVID-19-positive and (b) COVID-19-negative cough signals (shown across frequency bins and COVID status).

**Figure 5:**
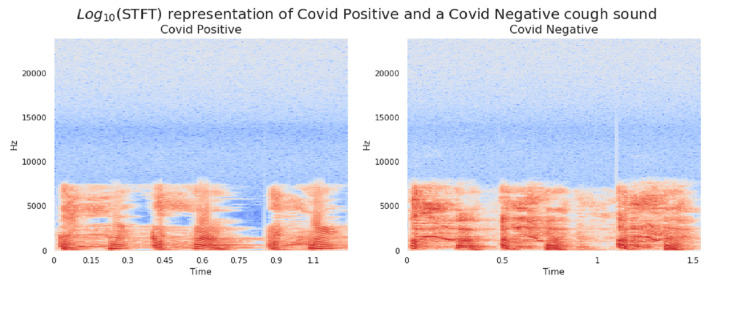
Log energy spectrum of typical cough sounds of people Left-hand side image: COVID-19-positive and Right-hand side image: COVID-19-negative statuses.

**Figure 6:**
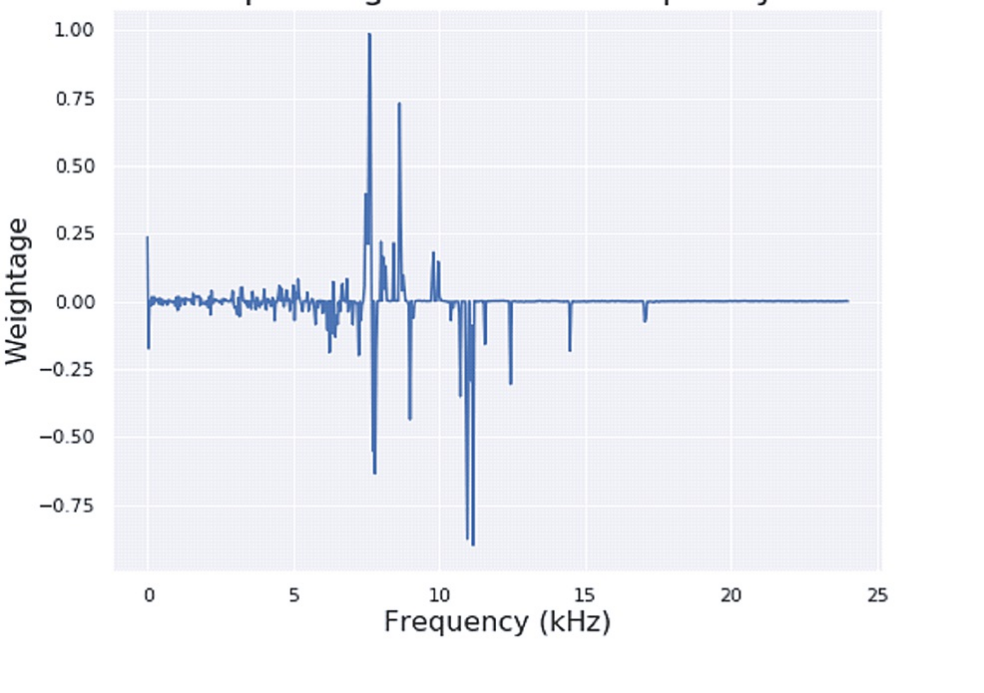
Weights learnt by the model for corresponding to various bins in a typical experiment

In the present study, Short Time Fourier Transform (STFT) feature representation was explored. The frame size of 25 milliseconds (1200 points FFT) and frameshift of 10 milliseconds (480 sample shift) were selected and the STFT was computed for all audio files. Each STFT frame of each audio file was assigned a target label (1 for +ve cases and 0 for -ve cases). This enabled the use of binary classifier models. Such models output a value of 1, if the presence of COVID-19 signatures in a frame is detected, and 0 otherwise. The output of such a model can be regarded as a probability of a positive case. Prediction of probability for a complete file (comprising of several STFT frames) was done by computing the mean of individual frames probabilities. Figure 4 shows the STFT of a COVID-19-positive and a non-COVID-19 cough. A clear difference between the spectrums in the two categories was identified. For each set of spectrums, the y-axis was adjusted to make the distinction clearer. The y-axis is adjusted to accommodate the energies in the spectral range (Figure 5). Figure 6 shows the mean energy spectrum (computed by averaging the STFT frames) of COVID-19-positive and COVID-19-negative cough sounds.

Logistic regression

In the present study, the logistic regression model was explored as the discriminator. Logistic regression is a simple yet powerful model. Its simplicity makes it easy to analyze and improve upon. This can guide us towards choosing the correct model architecture when deep neural networks are used. A logistic regression model is equivalent to a single-neuron artificial neural network with a sigmoid activation function. In the present case, the model took an STFT frame as input and computed the probability of it belonging to a COVID-positive category. Since the audio was sampled at 48 kHz and 1200 points FFT was used, there were 601 bins in each STFT frame (each bin having a width of 39.93 Hz). Training this model involved learning the weightage assigned to those frequency bins.

We trained the model using an adaptive moment estimation (ADAM) gradient descent algorithm. We used a learning rate of 0.001 Hz and a batch size of 16. The model was trained for 50 iterations in each experiment. While training, the cross-entropy loss function computed on the training data was minimized. The values of hyper-parameters, such as learning rate, batch size, and number of iterations were decided by observing the model performance on the validation set. In order to avoid overfitting associated with small sample sizes, L1 regularization with the coefficient of regularization set to 1e-3 was used. This too was decided based on observing performance on the validation set. The values of these hyperparameters can be further optimized to improve the overall performance.

Efficacy of the model

Using the procedure described in the previous section, 100 experiments were run on different partitions of training, validation, and test sets. The results of these experiments are summarized in Table 1. The table lists the mean and standard error values for loss functions and the accuracies attained at the end of each experiment. File-level accuracy (computed as a mean of frame probabilities) for the test sets is also listed.

**Table 1:** Loss and accuracy (mean ± std. error) for logistic regression model (100 experiments)

Training		Validation		Testing		File-level Testing
Loss (Mean ± SD)	Accuracy (%) (Mean ± SD)	Loss (Mean ± SD)	Accuracy (%) (Mean ± SD)	Loss (Mean ± SD)	Accuracy (%) (Mean ± SD)	Accuracy (%) (Mean ± SD)
1.002 ± 0.008	67.34 % ± 0.22	1.396 ± 0.055	58.57 ± 1.11	1.477 ± 0.055	57.82 ± 1.00	64.60± 1.79

In order to understand the mechanism of this model in distinguishing between the two classes, the weight values for the different frequencies were visualized and learned by the model (Figure 6). Since the energy values of STFT frames were found to be positive, a positive weight value contributes towards predicting samples as COVID-19 positive and vice versa. Figure 6 shows large spikes around 7.5 kHz, 7.8 kHz, 8.6 kHz and 11 kHz. It indicates that these frequencies are important for performing the classification. Another reason is that the energy values at higher frequencies (> 4 kHz) are smaller (Figure 6) compared to those at lower frequencies (<4 kHz), so they require larger weightage values to affect the decision.

**Figure 7:**
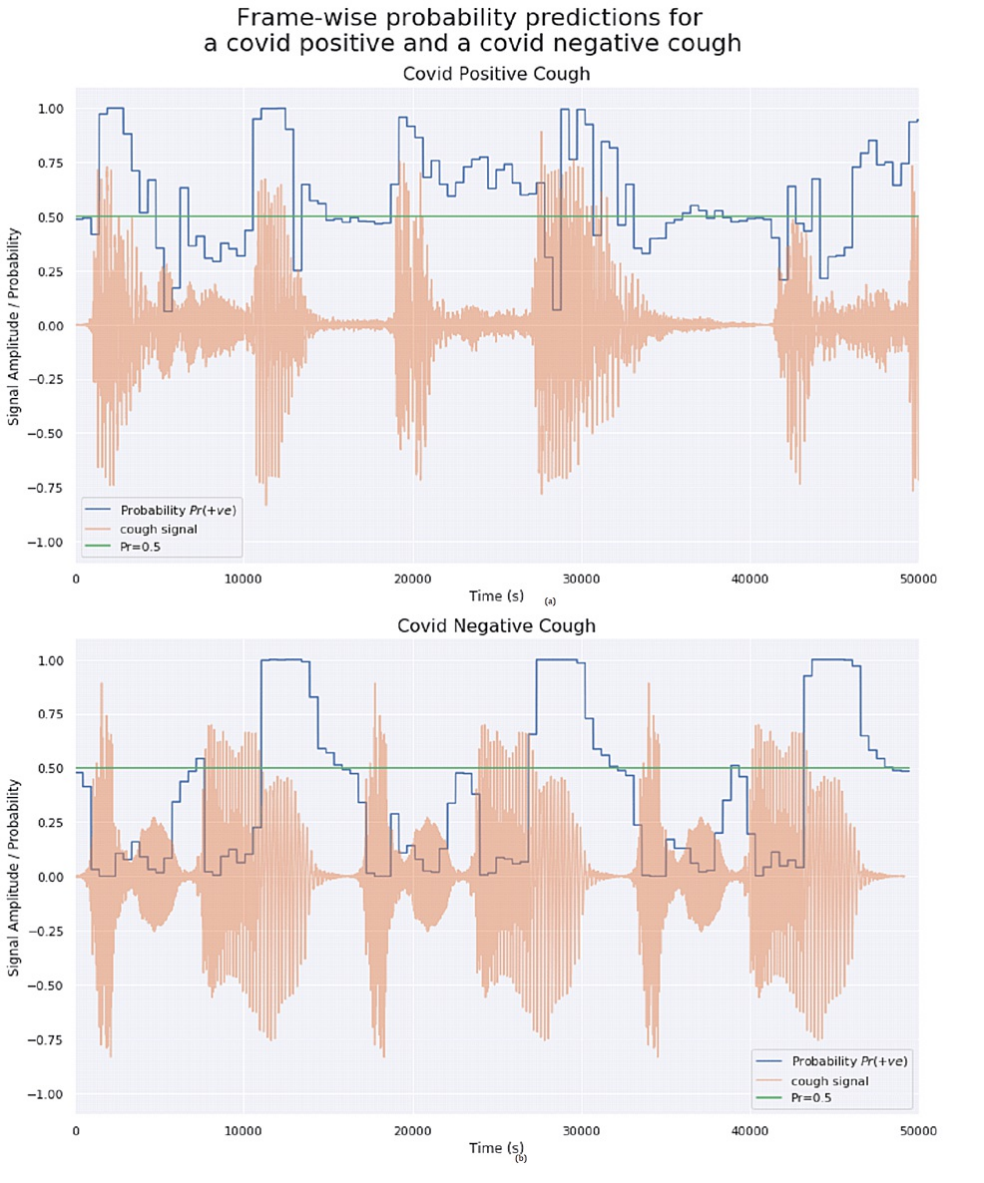
Frame-wise probabilities predicted by the logistic regression model (a) COVID-19-positive and (b) COVID-19-negative cough samples.

Figure 7 illustrates the probabilities assigned to each frame for a COVID-19-positive and a non-COVID-19 cough sample. A line marking probability value of 0.5 is also shown for easing comparison. It is noticed that the silent zones (low absolute amplitude values) have a probability close to 0.5. This shows that the model has correctly learned to attribute neutral weightages on silent portions in the audio. There are also several incorrectly classified frames in both cough samples. Notice that most of the incorrect classifications appear to be at or near lower signal amplitudes.

This fact and the neutral probability at silent regions motivated us to run another set of experiments where we modified the audio recordings. Here, audio frames where frame energy was less than one-third of the mean energy of the signal were eliminated. This thresholding algorithm is called speech activity detection and is typical in speech processing systems. With modified audios, our classification performance improved, the results of which are summarized in Table 2. While running this new set of experiments, all the other method parameters were kept the same.

**Table 2:** Result summary for modified audio experiments Note: Audio frames with energy less than a third of the mean energy were dropped.

Training		Validation		Testing		File-level Testing
Loss (Mean ± SD)	Accuracy (%) (Mean ± SD)	Loss (Mean ± SD)	Accuracy (%) (Mean ± SD)	Loss (Mean ± SD)	Accuracy (%) (Mean ± SD)	Accuracy (%) (Mean ± SD)
0.629 ± 0.002	66.20 % ± 0.12	0.789 ± 0.014	56.14 ± 0.64	0.640 ± 0.006	63.40 ± 0.56	74.80± 1.07

Since the energies in nearby frequency bins must be correlated with one another, we believe in applying dimensionality reduction to the performance. To explore this, the Principal Component Analysis (PCA) was used, but it did not cause any noticeable change in accuracy values, even though the loss values were slightly (2-3%) reduced.

## Discussion

Artificial intelligence is a new technique being increasingly popular in the medical field due to its varied applications in early diagnosis and management of various diseases. Researchers have used various AI-based techniques, such as machine and deep learning models, to detect diseases such as skin, liver, heart, Alzheimer's disease, etc. that need to be diagnosed early. Most AI models have been tried for chronic illnesses, but the application of AI in acute illness is limited to date [14].

COVID-19 is a real-world pandemic affecting people from most countries. Morbidity and mortality associated with COVID-19 have shaken the world and significant resources are diverted for its better management all over the world. An early diagnostic tool that is fast, accurate and useful for field screening is much needed for combating COVID-19. The diagnostics techniques available till now have been based on the detection of viral genes or human antibodies or viral antigen detection in the laboratory, among which viral gene detection by RT-PCR is the most widely used or radiological examination of the chest like HRCT. Few fast detection kits for COVID-19 are also available in the market [15]. This study is an attempt to develop an AI-based diagnostic tool for early detection of COVID-19 which can be utilized for field screening.

This tool is found to have an accuracy level of around 75% using vocal signatures in cough sounds. Also, there is a typical nature of human speech sounds. It is thought that the biological reasons for these frequencies being important for detecting COVID should be explored. The next logical step for improving the performance is to formulate the model such that it automatically learns to focus on important regions in the cough sounds. One candidate model for this is the Attention-based Neural Network [16]. It can be planned to explore its use in the future study.

Other researchers have worked in the field of AI and its use in medicine, but an AI-based diagnostic model using cough sounds for COVID-19 is unique. Earlier researchers have used techniques like the Boltzmann machine, K nearest neighbour (kNN), support vector machine (SVM), decision tree, logistic regression, fuzzy logic, and artificial neural network to diagnose diseases with remarkable accuracy. For example, a research study by Dabowsa et al. [17] used a backpropagation neural network in diagnosing skin disease to achieve the highest level of accuracy using real-world data collected from the dermatology department. Ansari et al. [18] used a recurrent neural network (RNN) to diagnose liver disease hepatitis virus and achieved 97.59%, while a feed-forward neural network achieved 100%. Owasis et al. [19] got a 97.057 area under the curve by using residual neural networks and long short-term memory to diagnose gastrointestinal disease. Khan and Member [20] introduced a computerized arrangement framework to recover the data designs. They proposed a five-phase machine learning pipeline that further arranged each stage in various sub-levels. They built a classifier framework alongside information change and highlighted choice procedures inserted inside a test and information investigation plan. Skaane et al. [21] enquired about the property of digital breast tomosynthesis on period and detected cancer in residents based on screening. They did a self-determining dual analysis examination by engaging ladies of 50-69 years and comparing full-field digitized mammography plus data-building tools with full-field digital mammography. Accumulation of the data-building tool resulted in a non-significant enhancement in sensitivity by 76.2% and a significant increase by 96.4%. Tigga and Garg [22] aimed to assess the diabetic risk among patients based on their lifestyle, daily routines, health problems, etc. They experimented on 952 collected via an offline and online questionnaire.

Recently AI has been tried in tuberculosis in India. Various AI and ML techniques were tried for understanding the epidemiology and disease spread of tuberculosis in India [23]. In this project, researchers have segregated data of tuberculosis co-morbidities (paediatric, geriatric, HIV) from multiple states and districts of India and correlated them with climate data, accustomed to bringing optimal precision to the prediction model in order to increase the efficacy of the system. A graphic user interface was provided to improve the comprehension of the expected solution to assist in geographically visualizing the control of Tuberculosis in India and its effects in the near future. Also, AI has been tried in India for the diagnosis of tuberculosis [24,25]. Reviewing these scopes in India, researchers have generated the idea of AI-based software for the diagnosis of COVID-19, a pandemic affecting worldwide, and worked towards the diagnosis of COVID-19 using AI-based software using cough sounds.

This study has developed an AI-based system for the diagnosis of COVID-19 based on cough sounds, but a few limitations of the study need mentioning. The present study was done on a very small dataset and extensive data needs to be gathered for improving confidence in the model. Due to limited data, higher complexity models such as neural networks could not be explored. Trying to fit neural networks with current data resulted in models that had test accuracies less than the current model. Data was collected in an uncontrolled environment, leading to data corruption with noise, which was then artificially removed through filtering. Better voice signatures for COVID-19 may be detected if the recording is done in an environment with very little background noise. Since all COVID-19-positive data was collected from a single source, there may be latent biases in the data. Due to these limitations of the current model and data, we envisage that further data collection, followed by detailed analysis and modeling will lead to models that can be used for patient screening in real-life situations.

## Conclusions

This study has presented the classification of cough sounds as belonging to the COVID-19 and non-COVID-19 categories based on the available dataset consisting of 117 cough samples belonging to COVID-19 and non-COVID-19 persons. Using STFT representation of audio signals and a logistic regression model, a classification accuracy of 74.80% ± 1.07% on files in the test set could be achieved. It is believed that the performance can be further improved by using different models and feature extraction algorithms.
